# Comparison of the Clinical Outcomes Between Gel Immersion and Underwater Endoscopic Mucosal Resection for Superficial Non‐ampullary Duodenal Epithelial Tumors (With Video)

**DOI:** 10.1002/deo2.70367

**Published:** 2026-06-22

**Authors:** Mitsuru Otsuka, Takuto Hikichi, Jun Nakamura, Tsunetaka Kato, Minami Hashimoto, Takumi Yanagita, Eisuke Kameoka, Rei Suzuki, Mitsuru Sugimoto, Hiroyuki Asama, Hiroshi Shimizu, Kento Osawa, Rei Ohira, Daiki Nemoto, Yuko Hashimoto, Masao Kobayakawa, Hiromasa Ohira

**Affiliations:** ^1^ Department of Gastroenterology Fukushima Medical University School of Medicine Fukushima Japan; ^2^ Department of Endoscopy Fukushima Medical University Hospital Fukushima Japan; ^3^ Department of Diagnostic Pathology Fukushima Medical University School of Medicine Fukushima Japan; ^4^ Medical Research Center Fukushima Medical University Fukushima Japan

**Keywords:** duodenal neoplasms, endoscopic mucosal resection, gel immersion endoscopy, superficial non‐ampullary duodenal epithelial tumors, underwater endoscopic mucosal resection

## Abstract

**Objective:**

Gel immersion endoscopic mucosal resection (GIEMR) has recently emerged as a novel technique for superficial non‐ampullary duodenal epithelial tumors (SNADETs). However, its clinical advantages over underwater EMR (UEMR) remain unclear. This study compared the short‐ and long‐term outcomes of GIEMR and UEMR for SNADETs.

**Methods:**

We retrospectively analyzed patients with SNADETs who underwent UEMR or GIEMR. The R0 resection rate, procedural duration, adverse events, and local recurrence were compared between the groups. Risk factors for non‐R0 resection were evaluated using logistic regression analyses.

**Results:**

Thirty‐two and 49 patients in the UEMR and GIEMR groups, respectively, were included. The R0 resection rate was higher in the GIEMR group than in the UEMR group (GIEMR vs. UEMR; 47.4 vs. 25.7%, *p* = 0.039). The procedural duration from liquid injection to completion of resection was shorter in the GIEMR group (4 vs. 6 min, *p* = 0.038). No delayed bleeding or perforation occurred in either group. Kaplan–Meier analysis demonstrated no significant difference in the cumulative local recurrence rate at 3 years between the groups (log‐rank test, *p* = 0.902). Tumor size and UEMR were identified as risk factors for non‐R0 resection in the univariate analysis; however, in the multivariate analysis, tumor size was identified as the only independent risk factor for non‐R0 resection (odds ratio, 1.172; 95% confidence interval, 1.050–1.309; *p* = 0.005).

**Conclusions:**

GIEMR achieved a higher R0 resection rate and shorter procedural duration than UEMR without increasing adverse events; longer follow‐up is warranted.

**Trial Registration:**

N/A (Only approval of the research protocol by an Institutional Reviewer Board of Fukushima Medical University).

## 1 Introduction

The detection rate of superficial non‐ampullary duodenal epithelial tumors (SNADETs) has increased, leading to a growing number of patients undergoing endoscopic mucosal resection (EMR) [1]. However, duodenal EMR remains technically challenging because of the limited endoscope maneuverability, thin duodenal wall, and high risk of perforation [2, 3].

To overcome these limitations of conventional EMR (CEMR), a novel technique termed underwater EMR (UEMR) was developed, in which the duodenal lumen is filled with saline, allowing the lesion to float and enabling snare resection without requiring submucosal injection [4, 5]. Subsequent studies have reported the feasibility and safety of UEMR for SNADETs and suggested its potential advantages over CEMR [6, 7, 8, 9, 10]. Previous studies have demonstrated the feasibility and safety of UEMR for SNADETs and suggested its advantages over conventional EMR [6, 7, 8, 9, 10].

More recently, gel immersion endoscopy has been introduced as a novel technique for securing a stable visual field during endoscopic procedures [11]. Following the introduction of a dedicated medical gel in Japan (VISCOCLEAR; Otsuka Pharmaceutical Factory, Tokushima, Japan), this technique has been increasingly applied to various endoscopic treatments [11, 12, 13, 14]. Gel immersion EMR (GIEMR), which uses gel instead of saline to fill the duodenal lumen, has also been applied to the treatment of SNADETs [15, 16, 17]. Because gel remains more stable within the lumen and is less likely to mix with intestinal fluids or blood, GIEMR may provide a clearer endoscopic view and facilitate safer and more accurate resection.

However, studies directly comparing GIEMR and UEMR remain limited and have mainly focused on short‐term outcomes [18, 19]. Moreover, the long‐term outcomes of GIEMR for SNADETs have not been sufficiently evaluated. Therefore, the present study aimed to compare the short‐ and long‐term outcomes of GIEMR and UEMR for SNADETs.

## 2 Methods

### 2.1 Study Design and Patients

This single‐center retrospective study included consecutive patients with duodenal lesions who underwent EMR at Fukushima Medical University Hospital between November 2016 and October 2024. Among these, patients with SNADETs treated with UEMR or GIEMR were enrolled.

The indication for UEMR or GIEMR was an endoscopically diagnosed adenoma or intramucosal carcinoma considered suitable for en bloc EMR. Lesions involving the major or minor papilla were excluded. Preoperative biopsy was not mandatory. Lesions pathologically diagnosed as non‐SNADETs after resection were excluded.

UEMR was performed as the standard EMR technique between November 2016 and December 2021, whereas GIEMR was adopted from January 2022 onward following the introduction of medical gel for endoscopic procedures in Japan.

This study was conducted in accordance with the Declaration of Helsinki and approved by the Institutional Review Board of Fukushima Medical University (Approval No. 2020–124). Written informed consent for the endoscopic procedures was obtained from all patients. An opt‐out approach for study participation was disclosed on the hospital website.

### 2.2 Endoscopic Procedures and Perioperative Management

All procedures were performed under sedation with propofol and pentazocine by endoscopists certified by the Japan Gastroenterological Endoscopy Society (JGES). Antithrombotic management followed the JGES guidelines [20, 21].

UEMR was performed using a therapeutic endoscope with a forward water‐jet function (GIF‐H290T, PCF‐Q260Ji, or PCF‐H290Ti; Olympus Medical Systems, Tokyo, Japan) and a high‐frequency electrosurgical unit (VIO 300D or VIO3; ERBE Elektromedizin, Tübingen, Germany). After saline infusion into the duodenal lumen, snare resection was performed without submucosal injection using a 10–25‐mm snare (SnareMaster; Olympus Medical Systems, or Captivator II; Boston Scientific, Marlborough, MA, USA). The mucosal defect was closed using endoclips, with additional polyglycolic acid sheet coverage when necessary.

Oral administration of a proton pump inhibitor or a potassium‐competitive acid blocker was initiated on the morning of the EMR. To assess for bleeding or perforation, a follow‐up endoscopy was performed on the day after EMR. Patients were kept fasting for 2 days, including the day of EMR, and if no adverse event was observed, oral intake was resumed on post‐EMR day 2. The patients were discharged on post‐EMR day 4.

**FIGURE 1:**
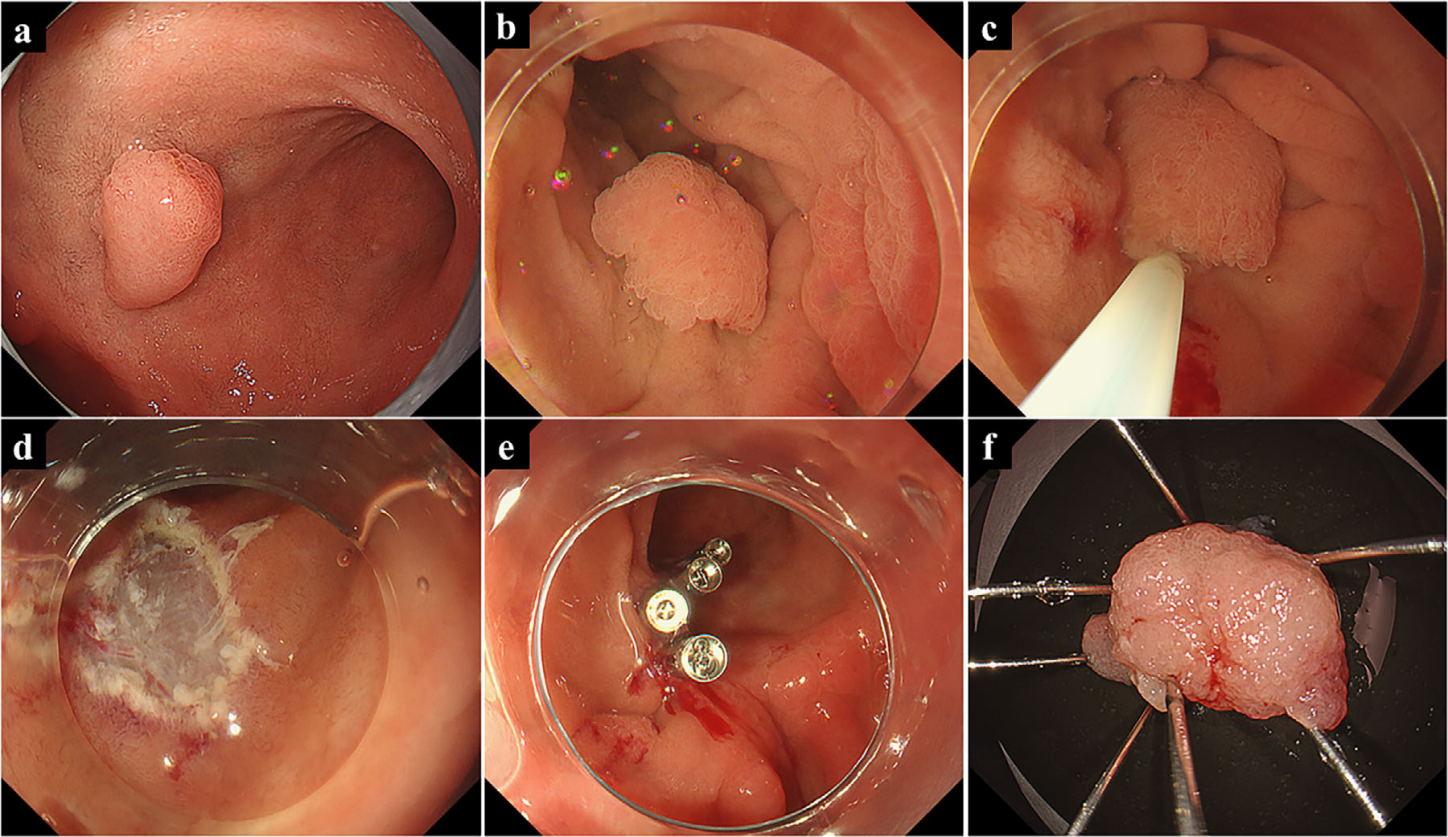
A representative case of superficial non‐ampullary duodenal epithelial tumor treated with gel immersion endoscopic mucosal resection. (a) A 10‐mm elevated lesion located in the duodenal bulb. (b) Filling the duodenal lumen with gel provides a more stable and clearer endoscopic view. (c) Snare resection performed after securing an adequate horizontal margin under gel immersion. (d) En bloc resection was successfully completed. (e) The post‐resection mucosal defect was completely closed using endoclips. (f) A resected specimen showing adenocarcinoma in an adenoma with negative horizontal and vertical margins.

**FIGURE 2:**
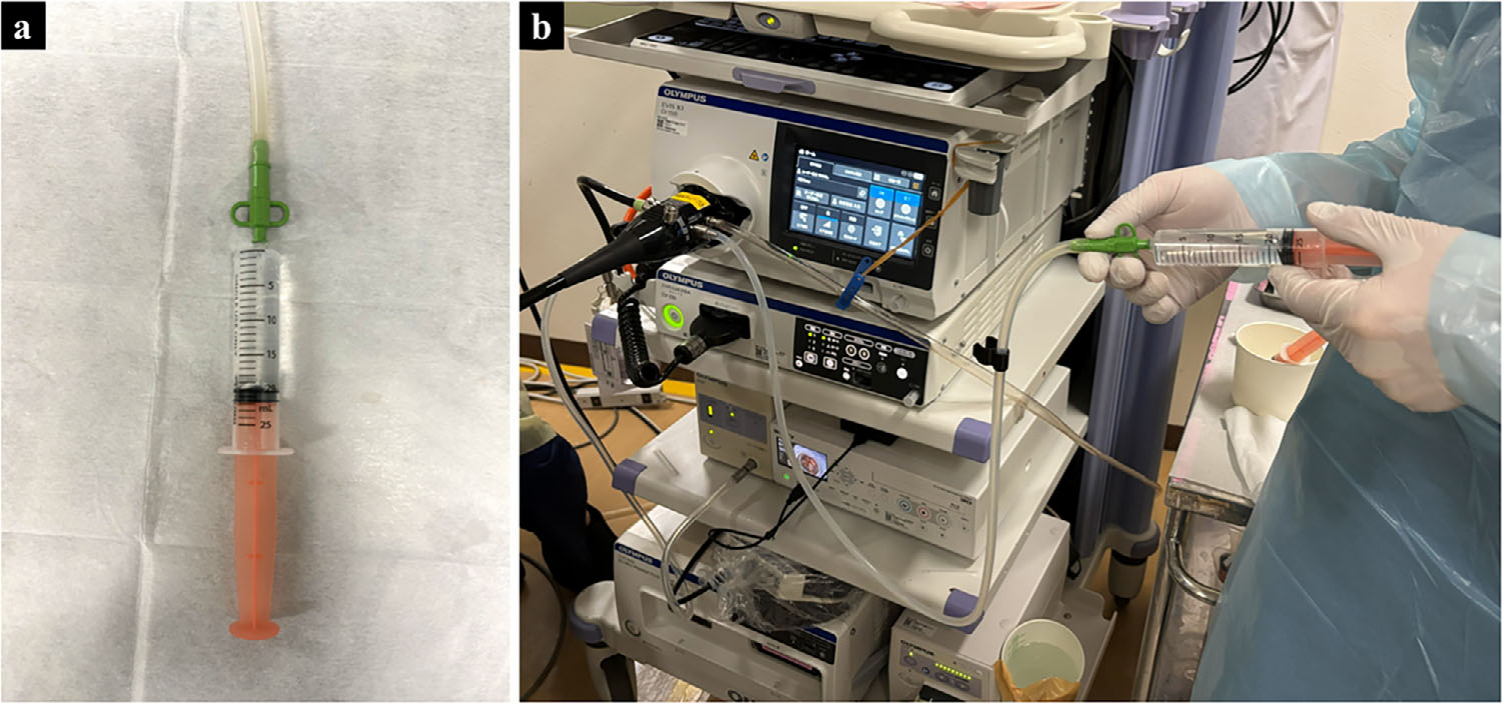
Additional gel injection during gel immersion endoscopic mucosal resection. (a) A syringe filled with gel connected to the tubing leading to the auxiliary water channel of the endoscope. (b) While the operator advances the snare through the working channel, the assistant simultaneously injects gel through the auxiliary water channel to maintain luminal filling.

In GIEMR, gel was used instead of saline, whereas the remaining procedure and perioperative management were identical to those of UEMR (Figure 1). When additional gel injection was required after snare insertion, gel was injected through the auxiliary water channel using a syringe (Figure 2 and Video S1) [13, 22].

### 2.3 Outcomes

The short‐term outcomes included the R0 resection rate, procedural duration, and procedure‐related adverse events. Univariate and multivariate logistic regression analyses were performed to identify factors associated with non‐R0 resection.

R0 resection was defined as en bloc resection with histologically negative horizontal and vertical margins. Non‐R0 resection was defined as positive or indeterminate horizontal or vertical margins. Resected specimens were serially sectioned at 2‐mm intervals and evaluated histopathologically using hematoxylin and eosin staining. When evaluating the horizontal margin was difficult, additional sectioning was performed at the discretion of the pathologist. In piecemeal resections, specimens were reconstructed to estimate tumor size.

The total procedural duration was defined as the interval from scope insertion to scope withdrawal and subdivided into the following three phases: 1) scope insertion to liquid injection, 2) liquid injection to completion of resection, and 3) completion of resection to scope withdrawal. The second interval was defined as the main procedural duration.

Procedure‐related adverse events included delayed bleeding, intraoperative perforation, delayed perforation, aspiration pneumonia, and procedure discontinuation due to hemodynamic or respiratory instability. Intraoperative bleeding was defined as bleeding requiring clips or hemostatic forceps. Delayed bleeding was defined as hematemesis or melena requiring endoscopic hemostasis. Intraoperative perforation was defined as either direct visualization of extraluminal structures during the procedure or post‐procedural free air detected on computed tomography (CT). Delayed perforation was defined as newly developed abdominal pain with free air on CT after completion of the procedure. Aspiration pneumonia was diagnosed based on suspected aspiration accompanied by fever and radiographic evidence of pneumonia.

Long‐term outcomes included local recurrence and survival in patients who underwent at least one follow‐up endoscopy after EMR. Surveillance endoscopy was performed 2–3 months after EMR and subsequently at 6‐ or 12‐month intervals. The follow‐up period was defined as the interval from EMR to the last follow‐up endoscopy. Survival was evaluated until December 31, 2025.

### 2.4 Statistical Analysis

Continuous variables are presented as medians with interquartile ranges, and categorical variables as numbers and percentages. Continuous variables were compared using the Mann–Whitney *U* test, whereas categorical variables were compared using the chi‐square test or Fisher's exact test. Local recurrence was analyzed using the Kaplan–Meier method and compared using the log‐rank test.

To identify factors associated with non‐R0 resection, univariate logistic regression analysis was initially performed for clinically relevant variables, followed by multivariate logistic regression analysis using a forced‐entry method. Lesion location was excluded from the multivariate model because of the imbalance in case distribution and the limited sample size. Odds ratios (ORs) and 95% confidence intervals (CIs) were calculated.

All statistical analyses were performed using IBM SPSS Statistics version 30.0.0 (IBM Corp., Armonk, NY, USA). A two‐sided *p*‐value <0.05 was considered statistically significant.

## 3 Results

### 3.1 Patient and Lesion Characteristics

**FIGURE 3:**
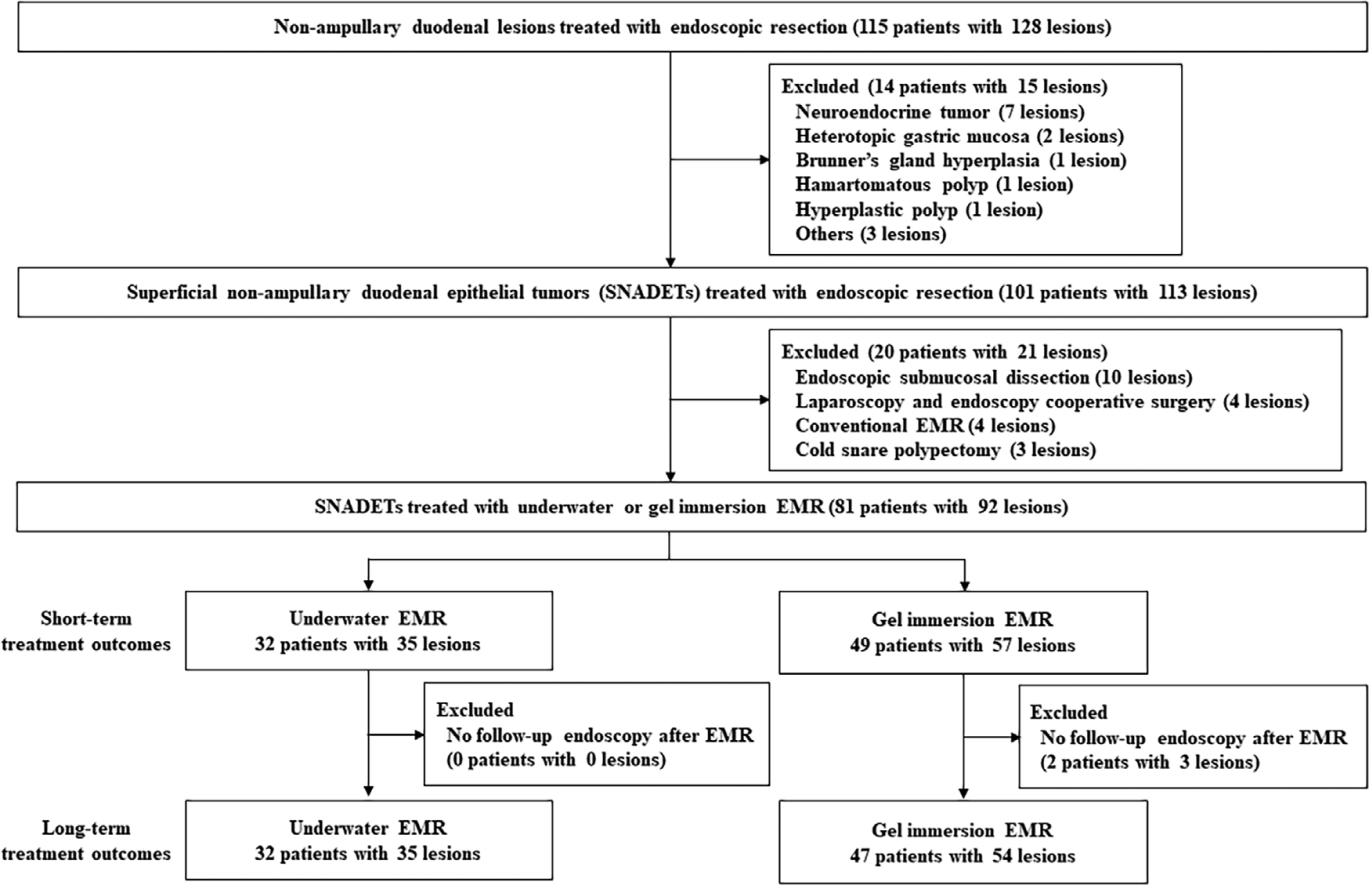
Study flowchart. A flowchart illustrating the patient selection process and study design. EMR, endoscopic mucosal resection; UEMR, underwater endoscopic mucosal resection; GIEMR, gel immersion endoscopic mucosal resection.

During the study period, 115 patients underwent EMR for duodenal lesions. After applying the exclusion criteria, 81 patients with 92 SNADETs treated by UEMR or GIEMR were included in the short‐term outcome analysis (Figure 3). For the long‐term outcome analysis, 79 patients with 89 lesions who underwent at least one follow‐up endoscopy were included.

The patient and lesion characteristics are summarized in Table 1. No significant differences were observed between the UEMR and GIEMR groups, except for operator distribution (*p* = 0.002). All procedures were performed by seven endoscopists, and the endoscopic experience and case distribution of each operator are shown in Table S1.

**TABLE 1:** Characteristics of the lesions and patients with superficial non‐ampullary duodenal epithelial tumors who underwent underwater endoscopic mucosal resection (UEMR) or gel immersion endoscopic mucosal resection (GIEMR).

Patient characteristics (*n* = 81)			
	UEMR (*n* = 32)	GIEMR (*n* = 49)	*p*‐Value
Age (years), median (IQR)	68.5 (63.5–72)	72 (60–76)	0.384
Sex, male, *n* (%)	25 (78.1)	32 (65.3)	0.217
Anticoagulation use, *n* (%)	2 (6.3)	4 (8.2)	1.000
Antiplatelet use, *n* (%)	3 (9.4)	0	0.058
Concomitant anticoagulant and antiplatelet therapy, *n* (%)	0	1 (2.0)	1.000

Lesion characteristics (*n* = 92)			
	UEMR (*n* = 35)	GIEMR (*n* = 57)	*p*‐Value
Location, *n* (%)			0.235
Bulb	6 (17.1)	7 (12.3)	
Second portion	25 (71.4)	48 (84.2)	
Third portion	4 (11.4)	2 (3.5)	
Macroscopic type, *n* (%)			0.123
Elevated	31 (88.6)	43 (75.4)	
Depressed	4 (11.4)	14 (24.6)	
Preoperative biopsy, *n* (%)	28 (80.0)	46 (82.1)	0.799
Operator, *n* (%)			0.002
A	12 (34.3)	12 (21.1)	
B	7 (20)	6 (10.5)	
C	1 (2.9)	0	
D	11 (31.4)	11 (19.3)	
E	3 (8.6)	9 (15.8)	
F	1 (2.9)	3 (5.3)	
G	0	16 (28.1)	

Abbreviations: GIEMR, gel immersion endoscopic mucosal resection; IQR, interquartile range; UEMR, underwater endoscopic mucosal resection.

### 3.2 Short‐term Outcomes of EMR

The short‐term outcomes are summarized in Table 2. The en bloc resection rate did not differ between the UEMR and GIEMR groups (82.9% vs. 82.5%, *p* = 0.961). However, the R0 resection rate was higher in the GIEMR group than in the UEMR group (47.4% vs. 25.7%, *p* = 0.039).

**TABLE 2:** Comparison of the treatment outcomes between underwater endoscopic mucosal resection (UEMR) or gel immersion endoscopic mucosal resection (GIEMR) for superficial non‐ampullary duodenal epithelial tumors.

	UEMR (*n* = 35)	GIEMR (*n* = 57)	*p‐*Value
Specimen size (mm), median (IQR)	16 (11–22)	15 (11–18)	0.449
Tumor size (mm), median (IQR)	10 (7–17)	10 (6–12)	0.176
Tumor size ≤ 20 mm, *n* (%)	31 (88.6)	54 (94.7)	0.421
Pathological diagnosis, *n* (%)			
Adenoma	30 (85.7)	51 (89.5)	0.742
Adenocarcinoma	5 (14.3)	6 (10.5)	
En bloc resection, *n* (%)	29 (82.9)	47 (82.5)	0.961
Horizontal margin, *n* (%)			
Negative	9 (25.7)	27 (47.4)	0.039
Unclear	8 (22.9)	13 (22.8)	
Positive	18 (51.4)	17 (29.8)	
Vertical margin, *n* (%)			
Negative	32 (91.4)	55 (96.5)	0.365
Unclear	3 (8.6)	2 (3.5)	
Positive	0	0	
R0 resection, *n* (%)	9 (25.7)	27 (47.4)	0.039
Complete closure of the mucosal defect after resection, *n* (%)	35 (100)	55 (96.5)	0.523
Procedural duration			
Duration from scope insertion to scope withdrawal (min), median (IQR)	41.0 (27.5–51.5)	27.0 (21–35)	0.005
Duration from scope insertion to liquid injection (min), median (IQR)	3.5 (2.0–5.5)	3.0 (2.0–4.0)	0.248
Duration from liquid injection to completion of resection (min), median (IQR)	6.0 (4.0–9.5)	4.0 (3.0–8.0)	0.038
Duration from resection to scope withdrawal (min), median (IQR)	25.5 (16.0–39.5)	18.0 (12.0–23.0)	0.007
Procedure‐related adverse event, *n* (%)			
Intraoperative perforation	0	0	―
Intraoperative bleeding^*^	3 (8.6)	4 (7.0)	1.000
Delayed perforation	0	0	―
Delayed bleeding	0	0	―
Aspiration pneumonia	1 (2.9)	0	0.380
Endoscopic follow‐up duration (months), median (IQR)	49.5 (26.0–63.0)	14.5 (3.1–27.9)	<0.001

Abbreviations: GIEMR, gel immersion endoscopic mucosal resection; IQR, interquartile range; UEMR, underwater endoscopic mucosal resection.

* Intraoperative bleeding was defined as bleeding that required clips or hemostatic forceps for hemostasis.

The median total procedural duration was shorter in the GIEMR group (27 vs. 41 min, *p* = 0.005). The main procedural duration from liquid injection to completion of resection was also shorter in the GIEMR group (4 vs. 6 min, *p* = 0.038). In addition, the duration from completion of resection to scope withdrawal was shorter in the GIEMR group (18 vs. 25.5 min, *p* = 0.007).

Intraoperative bleeding requiring clips or hemostatic forceps occurred in three lesions (8.6%) in the UEMR group and four lesions (7.0%) in the GIEMR group (*p* = 1.000). One patient (2.9%) in the UEMR group developed aspiration pneumonia, which improved with conservative treatment. No delayed bleeding, intraoperative perforation, delayed perforation, or procedure discontinuation due to hemodynamic or respiratory instability occurred in either group.

### 3.3 Factors Associated With Non‐R0 Resection

The results of the logistic regression analyses are shown in Table 3. In the univariate analysis, tumor size (p = 0.003) and UEMR (p = 0.042) were associated with non‐R0 resection. In the multivariate analysis, only tumor size remained an independent risk factor for non‐R0 resection (odds ratio, 1.172; 95% confidence interval, 1.050–1.309; p = 0.005).

**TABLE 3:** Factors associated with non‐R0 resection based on the results of the univariate and multivariate analyses.

	Univariate analysis			Multivariate analysis		
Variables	Odds ratio	95% CI	*p*‐Value	Odds ratio	95% CI	*p*‐Value
Location						
Bulb	Ref	―	―			
Second portion	0.948	0.282–3.185	0.932			
Third portion	1.250	0.164–9.538	0.830			
Macroscopic type						
Elevated	Ref	―	―	Ref	―	―
Depressed	0.761	0.268–2.156	0.607	1.649	0.486–5.597	0.423
Preoperative biopsy	1.460	0.505–4.223	0.485	0.811	0.229–2.865	0.745
Tumor size	1.154	1.050–1.267	0.003	1.172	1.050–1.309	0.005
Procedure						
GIEMR	Ref	―	―	Ref	―	―
UEMR	2.600	1.037–6.519	0.042	2.197	0.797–6.058	0.128

Abbreviations: CI, confidence interval; GIEMR, gel immersion endoscopic mucosal resection; UEMR, underwater endoscopic mucosal resection.

Subgroup analysis according to tumor size is shown in Table 4. The R0 resection rate decreased with increasing tumor size and was 0% for lesions >20 mm in both groups.

**TABLE 4:** The R0 resection rates according to tumor size.

Tumor size	Overall	UEMR (*n* = 35)	GIEMR (*n* = 57)
≤10 mm, % (*n*)	48.1 (25/52)	31.6 (6/19)	57.6 (19/33)
>10 to ≤20, % (*n*)	33.3 (11/33)	25.0 (3/12)	38.1 (8/21)
>20, % (*n*)	0 (0/7)	0 (0/4)	0 (0/3)

Abbreviations: GIEMR, gel immersion endoscopic mucosal resection; UEMR, underwater endoscopic mucosal resection.

### 3.4 Long‐term Outcomes After EMR

**FIGURE 4:**
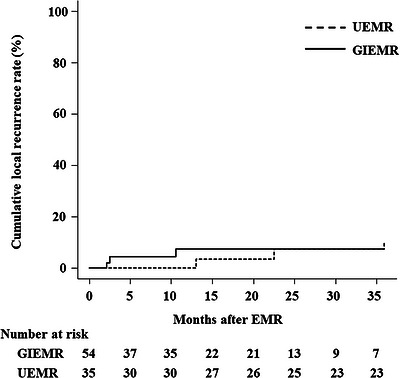
Cumulative incidence of local recurrence after endoscopic mucosal resection. The follow‐up duration was shorter in the GIEMR group. UEMR, underwater endoscopic mucosal resection; GIEMR, gel immersion endoscopic mucosal resection.

The Kaplan–Meier curves for local recurrence after EMR are shown in Figure 4, and the details of recurrent cases are summarized in Table 5. The cumulative local recurrence rate did not differ between the UEMR and GIEMR groups (log‐rank test, p = 0.902). However, the median endoscopic follow‐up duration was shorter in the GIEMR group than in the UEMR group (14.5 vs. 49.5 months, *p* < 0.001).

**TABLE 5:** Details of the recurrent cases occurring after endoscopic resection.

Procedure	Tumor size (mm)	Pathological diagnosis	Resection pattern	Resection status	Time to recurrence after resection (months)	Additional treatment
UEMR	10	Adenoma	En bloc	non‐R0	63.1	CFP
UEMR	4	Adenoma	En bloc	R0	36	UEMR
UEMR	20	Adenoma	Piecemeal	non‐R0	22.6	CFP
UEMR	16	Adenoma	Piecemeal	non‐R0	13.1	UEMR
GIEMR	25	Adenoma	Piecemeal	non‐R0	2.5	CFP
GIEMR	N/A^*^	Adenoma	Piecemeal	non‐R0	10.6	GIEMR
GIEMR	10	Adenoma	En bloc	non‐R0	2.1	CFP

Abbreviations: CFP, cold forceps polypectomy; GIEMR, gel immersion endoscopic mucosal resection; N/A, not applicable; UEMR, underwater endoscopic mucosal resection.

* Tumor size could not be evaluated due to multiple‐piecemeal resection.

During the study period, four recurrent lesions in the UEMR group and three recurrent lesions in the GIEMR group were identified. All recurrent lesions were successfully treated with additional endoscopic therapy, with no recurrence after retreatment. During follow‐up, one patient in the UEMR group died of ovarian cancer, and one patient in the GIEMR group died of cardiogenic pulmonary edema. No deaths were attributable to SNADETs in either group.

## 4 Discussion

This study demonstrated that GIEMR for SNADETs was associated with a higher R0 resection rate and shorter procedural duration than UEMR, without increasing procedure‐related adverse events. In addition, no significant difference in local recurrence or disease‐specific survival was observed between the two techniques during the available follow‐up period.

Previous comparative studies have also reported higher R0 resection rates with GIEMR than with UEMR [18, 19]. Consistent with these findings, the present study demonstrated superior histological resection quality with GIEMR in a larger cohort with longer follow‐up. Achieving R0 resection during duodenal EMR is technically challenging because the narrow lumen, abundant mucosal folds, and thin duodenal wall often obscure the lateral tumor margins and hinder precise snaring. In UEMR, saline may mix with intestinal fluids, bile, or blood, resulting in deterioration of the visualization. In contrast, gel remains stable within the lumen because of its viscoelastic properties, thereby facilitating clearer visualization and more accurate snaring with sufficient horizontal margins [16, 19, 23].

The overall R0 resection rates in the present study were lower than those reported previously [18, 19]. Several factors may explain this discrepancy. First, unlike previous feasibility studies performed by a limited number of expert endoscopists, the present study included procedures performed by seven JGES‐certified endoscopists with varying levels of experience, better reflecting real‐world clinical practice. Second, the definition of R0 resection differed between studies. In the present study, R0 resection was strictly defined as histologically negative horizontal and vertical margins without allowance for thermal burning effects.

Tumor size was identified as the only independent risk factor for non‐R0 resection in the multivariate analysis. In the subgroup analysis, the R0 resection rate decreased with increasing tumor size and was 0% for lesions >20 mm in both groups. Previous studies have similarly demonstrated lower R0 resection rates with EMR‐based techniques for larger SNADETs [10, 24]. These findings suggest that, regardless of whether saline or gel is used, EMR‐based techniques may be insufficient for lesions >20 mm, for which ESD should be considered to achieve curative resection.

GIEMR also shortened the procedural duration. Consistent with previous studies [18, 19], the duration from liquid injection to completion of resection was shorter in the GIEMR group. In addition, the duration from completion of resection to scope withdrawal was also shorter with GIEMR. Although the frequency of intraoperative bleeding did not differ between the groups, the shorter post‐resection phase observed in the GIEMR group may reflect the greater ease of hemostatic maneuvers under gel immersion. Gel suppresses blood dispersion and maintains a clearer visual field, thereby enabling prompt identification of bleeding points and more efficient hemostasis [25, 26].

Regarding safety, no delayed bleeding or perforation occurred in either group. Aspiration pneumonia developed in one patient in the UEMR group but not in the GIEMR group. Previous studies have suggested that retrograde saline flow during UEMR may contribute to aspiration risk [27]. The higher viscosity of gel may reduce proximal reflux into the esophagus and pharynx, potentially lowering the risk of aspiration‐related complications. In addition, the shorter procedural duration with GIEMR may reduce sedation‐related respiratory and hemodynamic instability.

No significant difference in cumulative local recurrence was observed between the two groups during follow‐up. However, because GIEMR was introduced more recently, the median follow‐up duration was substantially shorter in the GIEMR group. Therefore, the apparent similarity in recurrence outcomes should be interpreted cautiously, and further studies with longer follow‐up are required to clarify the long‐term durability of GIEMR. All recurrent lesions were successfully treated endoscopically, and no disease‐specific deaths occurred. Although most recurrences after duodenal EMR reportedly occur at the first follow‐up endoscopy [28], several recurrent lesions in the present study were detected during later surveillance. Because complete mucosal closure is frequently performed after duodenal EMR, residual lesions may occasionally be difficult to identify immediately after treatment. Therefore, long‐term surveillance remains important after EMR for SNADETs.

This study has several limitations. First, this was a retrospective single‐center study with a limited sample size. Second, UEMR and GIEMR were performed during different time periods; therefore, a learning‐curve effect could not be excluded. Third, the follow‐up duration was substantially shorter in the GIEMR group because of the more recent introduction of this technique. Consequently, the recurrence outcomes of GIEMR may not yet fully reflect its long‐term durability, and comparisons of long‐term recurrence between the two techniques should be interpreted with caution. Fourth, the saline or gel volume was not quantified. Finally, all procedures were performed by JGES‐certified endoscopists at a tertiary referral center, which may limit the generalizability of the findings.

In conclusion, GIEMR for SNADETs was associated with improved resection quality and shorter procedural duration compared with UEMR, without compromising safety. While recurrence outcomes appeared favorable, the relatively limited follow‐up of the GIEMR cohort warrants cautious interpretation of long‐term efficacy.

## Author Contributions

**Conceptualization**: M.O., T.H., and J.N.; **Methodology**: M.O., T.H., and J.N.; **Formal analysis**: M.O., T.H., J.N., and Y.H.; **Investigation**: T.K., M.H., T.Y., E.K., R.S., M.S., H.A., H.S., K.O., R.O., and D.N.; **Resources**: M.O., T.H., and J.N.; **Data curation**: M.O. and J.N. **Writing – original draft preparation**: T.Y., T.H., and J.N.; **Writing – review & editing**: T.K., M.H., T.Y., E.K., R.S., M.S., H.A., H.S., K.O., R.O., D.N., Y.H., M.K., and H.O. **Visualization**: M.O. and T.H.; **Supervision**: M.K. and H.O.; **Project administration**: T.Y. and T.H.

## Ethics Statement

**Approval of the research protocol by an Institutional Reviewer Board**: This retrospective study complied with the guidelines stipulated in the Declaration of Helsinki and was approved by the Institutional Review Board of Fukushima Medical University (Approval No. 2020–124: August 17, 2020).

## Consent

The requirement for obtaining informed consent for study participation (use of medical records) was waived in accordance with the Ethical Guidelines for Medical and Biological Research Involving Human Subjects in Japan, with an opt‐out notice posted on the hospital's website. Written informed consent for all endoscopic procedures was obtained from each patient.

## Conflicts of Interest

The authors declare no conflicts of interest.

## Funding

The authors have nothing to report.

## Supporting information




**Supporting Table 1**: Operator experience and case distribution for superficial non‐ampullary duodenal epithelial tumors treated by UEMR or GIEMR.

**Supporting Video 1**: Gel immersion endoscopic mucosal resection for a superficial non‐ampullary duodenal epithelial tumor.

## Data Availability

All data generated or analyzed during this study are included in this article. Further inquiries can be directed to the corresponding author.

## References

[1] K. Goda , D. Kikuchi , Y. Yamamoto , et al., “Endoscopic Diagnosis of Superficial Non‐Ampullary Duodenal Epithelial Tumors in Japan: Multicenter Case Series,” Digestive Endoscopy 26, no. Suppl 2 (2014): 23–29, 10.1111/den.12268. 24750144

[2] Y. Yamasaki , N. Uedo , Y. Takeuchi , et al., “Current Status of Endoscopic Resection for Superficial Nonampullary Duodenal Epithelial Tumors,” Digestion 97, no. 1 (2018): 45–51, 10.1159/000484404. 29393159

[3] M. Kato , T. Kanai , and N. Yahagi , “Endoscopic Resection of Superficial Non‐Ampullary Duodenal Epithelial Tumor,” DEN Open 2, no. 1 (2022): e54, 10.1002/deo2.54. 35310765 PMC8828234

[4] K. F. Binmoeller , F. Weilert , J. Shah , et al., “Underwater EMR Without Submucosal Injection for Large Sessile Colorectal Polyps (With Video),” Gastrointestinal Endoscopy 75, no. 5 (2012): 1086–1091, 10.1016/j.gie.2012.01.009. 22365184

[5] K. F. Binmoeller , J. N. Shah , Y. M. Bhat , et al., “Underwater EMR of Sporadic Laterally Spreading Nonampullary Duodenal Adenomas (With Video),” Gastrointestinal Endoscopy 78, no. 3 (2013): 496–502.e1, 10.1016/j.gie.2013.04.197. 23642790

[6] G. Shibukawa , A. Irisawa , A. Sato , et al., “Endoscopic Mucosal Resection Performed Underwater for Nonampullary Duodenal Epithelial Tumor: Evaluation of Feasibility and Safety,” Gastroenterology Research and Practice 2018, no. 1 (2018): 7490961, 10.1155/2018/7490961. 30158967 PMC6109562

[7] K. Hirasawa , Y. Ozeki , A. Sawada , et al., “Appropriate Endoscopic Treatment Selection and Surveillance for Superficial Non‐Ampullary Duodenal Epithelial Tumors,” Scandinavian Journal of Gastroenterology 56, no. 3 (2021): 342–350, 10.1080/00365521.2021.1885067. 33382001

[8] H. Tanaka , Y. Urabe , H. Takemoto , et al., “Can Underwater Endoscopic Mucosal Resection Be an Alternative to Conventional Endoscopic Mucosal Resection for Superficial Non‐Ampullary Duodenal Epithelial Tumors?” DEN Open 4, no. 3 (2024): e312, 10.1002/deo2.312. 37927952 PMC10624252

[9] M. Furukawa , A. Mitoro , T. Ozutumi , et al., “Efficacy of Underwater Endoscopic Mucosal Resection for Superficial Non‐Ampullary Duodenal Epithelial Tumor,” Clinical Endoscopy 54, no. 3 (2021): 371–378, 10.5946/ce.2020.218. 33596634 PMC8182245

[10] M. Kato , Y. Takeuchi , S. Hoteya , et al., “Outcomes of Endoscopic Resection for Superficial Duodenal Tumors: 10 Years' Experience in 18 Japanese High‐Volume Centers,” Endoscopy 54 (2022): 663–670, 10.1055/a-1647-6033. 34496422

[11] T. Yano , D. Nemoto , K. Ono , et al., “Gel Immersion Endoscopy: A Novel Method to Secure the Visual Field During Endoscopy in Bleeding Patients (With Videos),” Gastrointestinal Endoscopy 83, no. 4 (2016): 809–811, 10.1016/j.gie.2015.09.009. 26463338

[12] H. Teshima , K. Yamashita , and S. Oka , “Case of Gel Immersion Endoscopy: Efficacy of Identification and Achieving Hemostasis for Diverticular Hemorrhage in the Sigmoid Colon,” Digestive Endoscopy 34, no. 4 (2022): 1442–1443, 10.1111/den.14172. 35191088

[13] T. Kato , T. Hikichi , and T. Yanagita , “Gel‐Immersion‐Assisted Endoscopic Injection Sclerotherapy Under Observation With Texture and Color Enhancement Imaging for Esophageal Varices,” Digestive Endoscopy 37, no. 8 (2025): 895–897, 10.1111/den.14836. 40180791

[14] O. Dohi , N. Iwai , H. Fukui , et al., “Gel Immersion Endoscopic Submucosal Dissection Using a Scissor‐Type Knife for Superficial Non‐Ampullary Duodenal Epithelial Tumors,” DEN Open 6, no. 1 (2026): e70157. 40589618 10.1002/deo2.70157 PMC12208102

[15] T. Yachida , H. Kobara , N. Tada , et al., “Endoscopic Mucosal Resection Under Gel Immersion for Superficial Nonampullary Duodenal Epithelial Neoplasms,” Endoscopy 54, no. 8 (2022): E435–E436, 10.1055/a-1757-1234. 34496441

[16] A. Miyakawa , T. Kuwai , T. Miyauchi , et al., “Gel Immersion Endoscopy‐Facilitated Endoscopic Mucosal Resection of a Superficial Nonampullary Duodenal Epithelial Tumor: A Novel Approach,” VideoGIE 6, no. 9 (2021): 422–426, 10.1016/j.vgie.2021.05.007. 34527843 PMC8431274

[17] T. Shimada , Y. Kanno , and K. Ito , “Tip‐in Gel Immersion Endoscopic Mucosal Resection With Partial Submucosal Injection for a Superficial Nonampullary Duodenal Epithelial Tumor on the Duodenal Angulus,” Digestive Endoscopy 37, no. 2 (2025): 209–210, 10.1111/den.14702. 39467066

[18] A. Miyakawa , T. Kuwai , Y. Sakuma , et al., “A Feasibility Study Comparing Gel Immersion Endoscopic Resection and Underwater Endoscopic Mucosal Resection for Superficial Nonampullary Duodenal Epithelial Tumors,” Endoscopy 55, no. 3 (2023): 261–266, 10.1055/a-1985-1235. 35970190 PMC9974333

[19] T. Yamashina , M. Shimatani , Y. Takahashi , et al., “Gel Immersion Endoscopic Mucosal Resection for Superficial Nonampullary Duodenal Epithelial Tumors May Reduce Procedure Time Compared With Underwater EMR (With Video),” Gastroenterology Research and Practice 2022, no. 1 (2022): 2040792, 10.1155/2022/2040792. 35756502 PMC9217606

[20] K. Fujimoto , M. Fujishiro , M. Kato , et al., “Guidelines for Gastroenterological Endoscopy in Patients Undergoing Antithrombotic Treatment,” Digestive Endoscopy 26, no. 1 (2014): 1–14, 10.1111/den.12183. 24215155

[21] M. Kato , N. Uedo , S. Hokimoto , et al., “Guidelines for Gastroenterological Endoscopy in Patients Undergoing Antithrombotic Treatment: 2017 Appendix on Anticoagulants Including Direct Oral Anticoagulants,” Digestive Endoscopy 30, no. 4 (2018): 433–440, 10.1111/den.13039. 29733468

[22] H. Hayashi , T. Kanno , T. Yano , et al., “Dialysis, Antithrombotics, and Lesion Location: Who Benefits Most From Gel Immersion Endoscopy in Gastric Endoscopic Submucosal Dissection?” Digestion ahead of print, August 16, 2025, 10.1159/000548018. PMC12503684 40820412

[23] H. Toyonaga , K. Takahashi , T. Kin , et al., “Gel Immersion Technique for the Examination and Treatment of an Ampullary Tumor,” Endoscopy 54, no. 3 (2022): E115–E116, 10.1055/a-1625-9876. 33784756

[24] N. Yahagi , M. Kato , Y. Ochiai , et al., “Outcomes of Endoscopic Resection for Superficial Duodenal Epithelial Neoplasia,” Gastrointestinal Endoscopy 88, no. 4 (2018): 676–682, 10.1016/j.gie.2018.05.022. 29753040

[25] T. Yano , T. Takezawa , K. Hashimoto , et al., “Gel Immersion Endoscopy: Innovation in Securing the Visual Field—Clinical Experience With 265 Consecutive Procedures,” Endoscopy International Open 9, no. 7 (2021): E1123–E1127, 10.1055/a-1468-4323. 34222638 PMC8216780

[26] Y. Morihisa , Y. Yabuuchi , Y. Tokutomi , et al., “Identification of Active Gastric Bleeding and Achievement of Endoscopic Hemostasis via Gel Immersion Endoscopy,” Endoscopy 57 (2025): E435–E436. 40389251 10.1055/a-2589-1469 PMC12088876

[27] Y. Yamasaki , N. Uedo , Y. Takeuchi , et al., “Underwater Endoscopic Mucosal Resection for Superficial Nonampullary Duodenal Adenomas,” Endoscopy 50 (2017): 154–158, 10.1055/s-0043-119413. 28962044

[28] F. Barbaro , L. G. Papparella , M. F. Chiappetta , et al., “Clinical Outcomes of Endoscopic Mucosal Resection for Large Superficial Nonampullary Duodenal Epithelial Tumor: A Single‐Center Study,” European Journal of Gastroenterology and Hepatology 37, no. 4 (2025): 439–445, 10.1097/MEG.0000000000002765. 39976048 PMC12416897

