# Supplementary material for: Comparison of the Clinical Outcomes Between Gel Immersion and Underwater Endoscopic Mucosal Resection for Superficial Non‐ampullary Duodenal Epithelial Tumors (With Video)
Source: DEN Open. 2026 Jun 22;7(1):e70367. doi: 10.1002/deo2.70367 (PMC13287325; doi:10.1002/deo2.70367)
Supplement: Supplementary file 1 — Supporting Table 1: Operator experience and case distribution for superficial non‐ampullary duodenal epithelial tumors treated by UEMR or GIEMR. [file DEO2-7-e70367-s001.docx]

**Supplementary Table S1. Operator experience and case distribution for superficial non-ampullary duodenal epithelial tumors treated by UEMR or GIEMR**

|  | UEMR (n = 35) | | GIEMR (n = 57) | |
| --- | --- | --- | --- | --- |
| Operator | Endoscopy experience at first procedure (years) | Cases, n (%) | Endoscopy experience at first procedure (years) | Cases, n (%) |
| A | 22 | 12 (34.3) | 26 | 12 (21.1) |
| B | 11 | 7 (20) | 15 | 6 (10.5) |
| C | 6 | 1 (2.9) | N/A | 0 |
| D | 6 | 11 (31.4) | 9 | 11 (19.3) |
| E | 5 | 3 (8.6) | 8 | 9 (15.8) |
| F | 7 | 1 (2.9) | 7 | 3 (5.3) |
| G | N/A | 0 | 6 | 16 (28.1) |

UEMR, underwater endoscopic mucosal resection; GIEMR, gel immersion endoscopic mucosal resection; N/A, not applicable
